# Research progress in different physical therapies for treating peripheral nerve injuries

**DOI:** 10.3389/fneur.2025.1508604

**Published:** 2025-04-07

**Authors:** Xiao-Lei Chu, Xiao-Xuan Zhao, Shuai-Yi Liu, Ya-Jie Li, Ning Ding, Min-Qi Liu, Qing-Wen Li, Qi Li

**Affiliations:** ^1^Department of Rehabilitation, Tianjin University Tianjin Hospital, Tianjin, China; ^2^Tianjin Key Laboratory of Exercise Physiology and Sports Medicine, Institute of Sport, Exercise and Health, Tianjin University of Sport, Tianjin, China

**Keywords:** peripheral nerve regeneration, ultrasound, electricity, photobiomodulation, aerobic exercise

## Abstract

Physical therapy is gaining recognition as an effective therapeutic approach in the realm of peripheral nerve injury (PNI) research. This article seeks to provide a comprehensive review of the latest advancements, applications, and mechanisms of action of four physical therapy modalities—ultrasound, electrical stimulation, photobiomodulation, and aerobic exercise—in the context of PNI. Ultrasound, characterized by its mechanical and thermal effects, is widely regarded as an effective non-invasive or minimally invasive method for neural modulation. Electrical stimulation therapy, a prevalent technique in PNI treatment, entails the application of electric currents to stimulate nerve and muscle tissues, thereby facilitating nerve regeneration and mitigating muscle atrophy. Photobiomodulation, a process that influences cell metabolism through the absorption of photon energy, is closely associated with neural regeneration in the field of rehabilitation medicine. Additionally, aerobic exercise, a popular form of physical activity, serves to enhance blood circulation and improve neuronal function. The article discusses various physical therapy methods for peripheral nerve injuries, including hyperbaric oxygen therapy, magnetic therapy, and biofeedback therapy, in addition to traditional approaches. Despite advancements, challenges in nerve injury treatment persist, such as the need for standardized treatment protocols, consideration of individual variations, and assessment of long-term effectiveness. Future research is needed to address these issues. In summary, this article offers theoretical and empirical evidence supporting the utilization of physical therapy in the management of PNI. This research aims to promote further research and clinical practice in this field, contributing to enhancing patient quality of life and recovery outcomes.

## 1 Introduction

Peripheral Nerve Injury (PNI) involves harm to nerves located outside the brain and spinal cord, typically resulting from direct trauma or indirect factors such as accidents, combat, or complications arising from illnesses (1). In Europe and America, traumatic peripheral nerve injuries affect hundreds of thousands of patients each year (2). In addition to a high incidence rate, PNI exhibits the following characteristics: slow growth, with regenerating nerves progressing at a rate of ~1 mm per day, and a complex direction of regeneration that frequently results in nerve misrouting and looping (3). This slow growth rate can cause the corresponding target organs to be in a state of denervation for a long time, leading to a certain degree of motor dysfunction, sensory dysfunction, and autonomic dysfunction, with a poor prognosis. Studies show that only 10%−25% (4) of those with nerve injuries fully recover their functions. Furthermore, in addition to inducing lesions at the site of trauma, It also results in abnormal communication between the central nervous system and the periphery, causing varying degrees of pathology, affecting mobility and bringing emotional and economic burdens (5).

PNI, due to tissue damage, suturing, and fixation, often leads to peripheral tissue edema and adhesions, and even muscle contractures and joint stiffness, ultimately resulting in severe impairment of limb function. Physical factor therapy, such as electrical, ultrasound, and photobiomodulation, can effectively alleviate these symptoms. Furthermore, various physical factor therapies also promote nerve regeneration to some extent, but their specific mechanisms of enhancement are currently unclear, making it difficult to accurately select the appropriate physical factor therapy and set the right parameters. In addition, aerobic exercise, as a safe and effective rehabilitation method, plays a significant role in facilitating the recovery from PNI. Similar to physical factor therapy, exercise therapy lacks standardized treatment protocols, particularly in terms of planned intensity, duration, and timing of initiation. Additionally, various other physical therapies, including magnetic therapy, such as magnetic therapy, hyperbaric oxygen therapy, and cryotherapy, have been utilized in the treatment of PNI with favorable outcomes.

**Figure 1:**
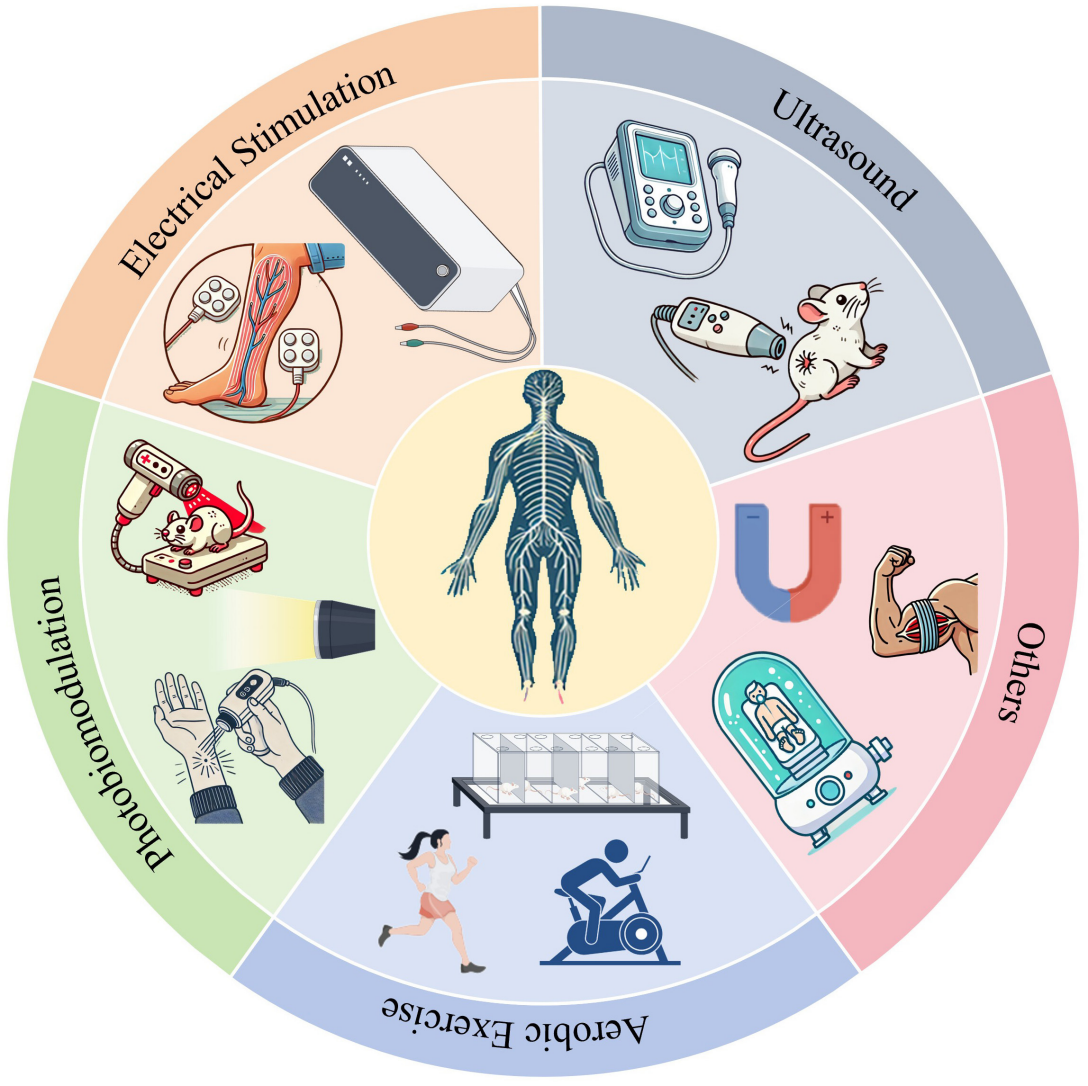
Management of peripheral nerve injuries through various physical therapy modalities.

The objective of this review is to comprehensively outline the advancements in research and potential mechanisms of various physical therapies for enhancing the functional rehabilitation of PNI. Specifically, this review will concentrate on the impacts of ultrasound, electrical stimulation, photobiomodulation, and other physical modalities, along with the role of aerobic exercise in PNI recovery. Ultrasound, characterized as a mechanical wave, possesses the ability to accurately transmit energy into tissues at significant depths in both spatial and temporal domains (6). Its thermal properties make it an advantageous modality for non-invasive or minimally invasive neuromodulation procedures. Electrical stimulation therapy, a common physical factor therapy in PNI, utilizes electrical currents to stimulate nerves and muscle tissues. This method can facilitate nerve regeneration and reduce muscle atrophy (7). Photobiomodulation is intricately associated with nerve regeneration due to its photochemical and photobiological mechanisms that influence cellular processes, as well as its capacity to stimulate the secretion of neurotrophic factors (8). Aerobic exercise, a prevalent form of physical activity, is known to enhance cardiovascular function and oxygen delivery, potentially mitigating muscle atrophy and spinal alterations following PNI (Table 1). This review also introduces other physical factor therapies used in PNI (Figure 1). In response to the shortcomings of various therapies and the current inadequacy of physiotherapy in the treatment of PNI, we propose a strategy of integrated physiotherapy, which we believe is promising in the treatment of PNI.

**Table 1:** The systemic alterations following PNI and the mechanisms through which aerobic exercise facilitates PNI recovery.

**Site**	**Systemic changes induced by PNI**	**Aerobic exercise mechanisms promoting PNI recovery**
Target organ (skeletal muscle)	Muscle atrophy. There may be a link between this phenomenon and the release of inflammatory cytokines and reactive oxygen species by M1 macrophages in damaged muscle tissue, resulting in myolysis (132).	Stimulates satellite cells to facilitate muscle hypertrophy (133).
Spinal dorsal root ganglia	Excessive discharge. This phenomenon may be attributed to the transmission of excitatory cytokines (50), by inflammatory cells, which subsequently activate spinal microglial cells (134).	It regulates autophagy through the AKT/mTOR signaling pathway, facilitating the polarization of spinal dorsal root ganglia cells post-nerve injury (94), which subsequently ameliorates sensory dysfunction. Additionally, it enhances the endogenous opioid system by inhibiting the activation of spinal dorsal horn glial cells, thereby mitigating neuropathic pain (105).
Brain	Changes in sensory motor cortex. This phenomenon may be attributable to BDNF produced by microglial cells, which induces cortical reorganization and subsequently leads to sensory dysfunction (130).	Enhances serotonin expression (106) and significantly mitigates the reduction in the primary somatosensory cortex, motor cortex regions, and hypothalamus (100).

## 2 Search strategy and selection criteria

We conducted a detailed search in the PubMed database using the following keywords: “peripheral nerve injury”, “peripheral nerve regeneration”, “functional recovery”, “mechanisms”, “ultrasound”, “electrical stimulation”, “photobiomodulation”, “low-intensity laser”, and “aerobic exercise”. Our search focused on articles published between 2019 and July 2024. However, to encompass literature of significant importance, we also included seminal works published prior to this period. After removing duplicates, we initially screened the titles and abstracts of each article. We then reviewed the full texts to exclude studies that did not pertain to peripheral nerve injury and physical therapy.

## 3 The impact of ultrasound on peripheral nerve injury

### 3.1 The application of ultrasound in peripheral nerve injury

Ultrasound (US) is a high-frequency sound wave that surpasses the human auditory threshold of 20 kHz and is frequently employed in medical imaging for safe and non-invasive clinical procedures (9). Furthermore, as a mechanical vibratory wave, the mechanical energy produced by US can be absorbed by biological tissues to achieve therapeutic purposes. In rehabilitation medicine, the commonly used frequencies are 800–1,000 kHz (10). The exploration of US as a therapeutic modality has roots in ancient times. In 1929 (11), the irradiation of a frog's sciatic nerve by US has been observed to induce minor twitches in the gastrocnemius muscle. This phenomenon has led scholars to investigate the potential of US in promoting the healing of fractures, tendons, ligaments, and other soft tissues. Within the realm of peripheral nerve regeneration, US is specifically referred to as Low-Intensity Pulsed Ultrasound (LIPUS). This modality holds promise for neural regulation (12, 13). In 1958, researchers (14) identified that high-intensity US possesses the ability to mitigate motor disorders through its thermal properties. This discovery underscored the considerable promise of high-intensity US's thermal effects for treating Parkinson's disease and chronic pain. Furthermore, studies have indicated that US may impact brain function, with evidence suggesting an inhibitory influence on visual cortex activity in felines. In 2008, Tyler et al. (15) conducted a study on the effects of LIPUS on neuronal activity. Their findings indicated that LIPUS has the capability to stimulate neuron and network activity remotely and non-invasively, implying that US can serve as an effective tool for modulating brain circuit activity from a distance. Several studies have examined the influence of US on PNI. Affonso conducted a study in 2002 (16) investigating the impact of pulsed US on rat sciatic nerve transection injuries, revealing that US stimulation led to accelerated nerve regeneration.

Research (17) shows that LIPUS treatment enhances functional performance in PNI. After 1 or 4 weeks of daily 5-min sessions at 1 MHz frequency, 140 mW/cm^2^ intensity, and a 20% duty cycle, the tibialis anterior muscle's CMAP rose by 21.88%, showing improved neuromuscular reinnervation. While myelin sheath thickness increased after 4 weeks compared to the control, there was no significant change in wet muscle weight ratio, suggesting LIPUS may have limited impact on muscle atrophy. Numerous research studies have employed the Sciatic Functional Index (SFI) as a tool for assessing the efficacy and recuperation of sciatic nerve function in rats subjected to models involving sciatic nerve injury (18, 19). The study revealed that the SFI was appreciably elevated in the LIPUS treatment group in comparison to the control group, demonstrating diverse recovery outcomes across different parameters. Selecting the right US parameters is vital for PNI treatment. Jiang et al. (20) found that a low-dose US (250 mW/cm^2^) was more effective than medium (500 mW/cm^2^) and high-dose (750 mW/cm^2^) US in treating sciatic nerve injury. The low-dose group exhibited longer axonal growth, thicker cross-sectional area, and myelin sheath, with consistent results across electron microscopy, toluidine blue staining, and more organized muscle fibers in Masson staining. This study shows that low-dose US enhances nerve regeneration better than higher doses. Low-intensity pulsed ultrasound at 0.3 W/cm^2^ boosts Schwann cell growth *in vitro* and is advised for early application on injured nerves, especially in low-serum environments, for optimal results. An animal study (21) showed that 140 mW/cm^2^ ultrasound significantly increased myelin fiber density by 26.59% and myelin cross-sectional area by 38% compared to controls, despite no change in axonal area.

### 3.2 Mechanisms of ultrasound therapy for peripheral nerve injury

**Figure 2:**
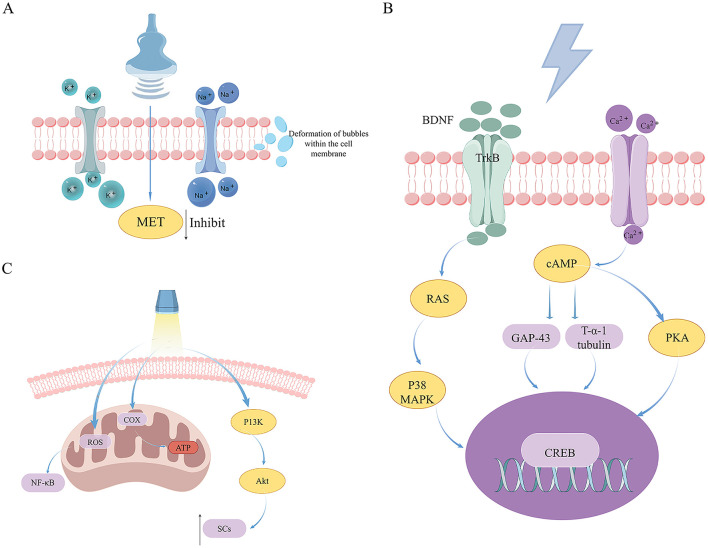
An examination of the mechanisms underlying US, ES, and PBM in the therapeutic management of peripheral nerve injuries. **(A)** The mechanisms of US therapy in treating PNI. **(B)** The mechanisms of ES therapy in treating PNI. **(C)** The mechanisms of PBM therapy in treating PNI.

The biological mechanisms of US therapy encompass mechanical effects, thermal effects, and the physicochemical effects induced by both (cavitation effects). Firstly, because nerve cell membranes contain numerous sensitive ion channels, when US vibration acts on nerve cells, it causes changes in membrane potential thereby affecting nerve signal conduction. Research findings suggest that US with varying parameters can selectively activate mechanically sensitive sodium and potassium channels, contributing to the diverse effects of US on the nervous system (22). Secondly, US on the body can generate a thermal effect, which is the result of acoustic energy converting into heat energy. Finally, the cavitation effect of US refers to the formation of microbubbles of gas in liquids (23), which subsequently oscillate or rupture under appropriate parameters, thereby affecting surrounding tissues or cells. Research on cell interactions suggests a Bilayer Sonophore (BLS) model, which proposes that US impacts intercellular communication by causing deformation of intracellular gas bubbles. This alteration may lead to changes in biochemical signaling pathways, ultimately affecting cell morphology and functionality (24). Using this model, it has been shown that LIPUS modulates cell motility and morphology through the inhibition of MET activation. These findings imply a reciprocal relationship between the intracellular MET signaling pathway and exposure to LIPUS (25) (Figure 2A).

In the context of PNI treatment in the US, the impact of US is predominantly observed in neuroglial cells, inflammatory cells, and NGF. Neuroglial cells play a vital role as mediators in the process of peripheral nerve regeneration, with particular emphasis on the research surrounding Schwann cells (SCs). In a study conducted by Crisci and Ferreira (16), it was determined that LIPUS stimulation increases the activity of SCs, thereby expediting the early restoration of myelin sheaths. Ren et al. (26) suggested that LIPUS enhances Schwann cell proliferation and nerve regeneration by activating the GSK-3β/β-catenin pathway. GSK-3β, a cytoplasmic kinase, influences developmental signaling and regulates Cyclin D1 transcription through β-catenin phosphorylation, facilitating SC proliferation. Nonetheless, the exact influence of GSK-3β on PNI remains ambiguous, as some research suggests that LIPUS facilitates axon regeneration by suppressing GSK-3β activity (27). It is hypothesized that targeted inhibition of GSK-3β can promote neurite outgrowth, while its overexpression may hinder neurite extension (28). Certain hypotheses suggest that nerve regeneration induced by LIPUS occurs via axonal mediation, without reliance on the proliferation of SCs. The utilization of LIPUS on dorsal root ganglion neurons has been shown to enhance axonal growth by a factor of two, potentially by stimulating the Netrin-1/DCC pathway (29). Subsequent to PNI, activated SCs can stimulate immune reactions; studies have demonstrated that LIPUS reduces the expression of cytokines involved in inflammation TNF-α and IL-6 in rats seven days following nerve crush injury, suggesting that LIPUS may attenuate pro-inflammatory reactions during the process of peripheral nerve regeneration (21). The results of the studies show that LIPUS has a significant inhibitory effect on the mature IL-1β in both *in vitro* and *in vivo* settings, as well as an increase in macrophage autophagy levels (30, 31). Furthermore, the research indicates that LIPUS significantly enhances the expression of M2 macrophage-associated genes while suppressing M1 macrophage-related genes, suggesting a potential role in regulating inflammation following PNI. Neurotrophic factors have been shown to protect nerve, axon regeneration, and myelinogenesis following PNI (32, 33). Augmenting the efficacy of NGF could serve as a promising therapeutic strategy for promoting nerve regeneration; studies indicate (34) LIPUS significantly increases NGF-induced neurite lengths, attributed to LIPUS-enhanced ERK1/2 and cAMP activates cyclic AMP response element-binding protein CREB phosphorylation induced by NGF, as well as enhanced expression of Trx-1. The findings indicate that LIPUS may augment NGF-induced neuronal growth by activating the ERK1/2-CREB-Trx-1 signaling pathways through mechanical means.

In summary, LIPUS aids PNI recovery through complex mechanisms such as enhancing glial cell activity, modulating immune responses, and activating nerve growth factors. These actions offer new therapeutic insights for nerve repair. However, more research and clinical trials are necessary to fully assess its clinical effectiveness and optimal application.

## 4 The impact of electrical stimulation on peripheral nerve injury

### 4.1 The application of electrical stimulation in peripheral nerve injury

Electrical stimulation (ES) is a viable and efficacious therapeutic modality for PNI, demonstrating considerable potential for promoting nerve regeneration. Extensive research has been conducted on ES as a physical therapy for PNI, with historical roots dating back to the early nineteenth century when it's potential to mitigate functional deficits in affected organs was recognized. In 1841 (35), the study revealed that ES had the capability to elicit muscle contractions in denervated frog legs and mitigate muscle atrophy. Many animal experiments have shown that ES can promote nerve regeneration. In 1952, Hoffman et al. (36) applied ES at frequencies ranging from 50 to 100 Hz to the sciatic nerve of rats with partially severed L5 nerve roots, resulting in an observed increase in axonal sprouting velocity following ES application. It was hypothesized by researchers that ES triggered retrograde signals in neuronal cell bodies, resulting in an expedited axoplasmic synthesis and improved axonal sprouting. ES has demonstrated efficacy in promoting nerve axon regeneration in animal models, and more recently, researchers have successfully translated this approach to human populations with peripheral nerve injuries, yielding favorable therapeutic results. In 2009, Gordon et al. (37) treated patients with median nerve injuries postoperatively with ES. A notable increase in axon regeneration was noted at the 6–8 month mark in comparison to the control group, accompanied by enhancements in MUNE, motor latency, and sensory nerve conduction velocity. In a study conducted in 2020, Power (38) observed that postoperative ES led to improvements in electromyographic indices, grip strength, and pinch strength among patients diagnosed with cubital tunnel syndrome.

With the progression of research into ES for the treatment of PNI, uncertainties associated with ES parameters have also become evident. A 1 h, 20 Hz non-invasive electrical stimulation (ES) is a widely used treatment in animal studies and clinical trials, known for its therapeutic benefits (39, 40). Additionally, Brushart et al. (41) found that this stimulation improved nerve regeneration at the junction but didn't speed up growth. Lu et al. (42) examined transcutaneous ES at 1, 2, 20, and 200 Hz with 1 mA intensity, finding that higher frequencies reduced regeneration. In the 2 Hz group, rats had smaller axonal areas, thicker myelin, higher density, and more blood vessels. Furthermore, the timing and length of ES are crucial elements; nonetheless, research using mouse models (43) showed no notable difference in results between 1 h and 10 min ES sessions (16 Hz, 100 μs). Roh et al. (43) further substantiated these findings, indicating that a 10 min duration of ES is adequate to elicit therapeutic effects comparable to those observed with 1 h ES in mouse models of nerve transection and repair. This data indicates that axonal regrowth might not solely depend on the length of ES, but also on other elements, possibly the number of triggered. Traditionally, ES has been administered immediately following surgical procedures. However, recent investigations have explored the application of ES in preoperative contexts to augment its efficacy. This preoperative application of ES is referred to as Conditioned Electrical Stimulation (CES) (44). In contrast, postoperative ES (PES) has been shown to facilitate nerve regeneration by minimizing misdirection at the neural junction. CES accelerates axonal extension. Senger et al. (45) compared one-week CES with immediate PES before nerve repair surgery and found that CES-treated axons extended further than those in the PES group, with significant improvements in sensory recovery and motor reinnervation, possibly due to reduced inflammation. In 2018, Barber et al. (46) demonstrated that intraoperative 1 h sessions of 20 Hz ES significantly enhanced shoulder function in patients with spinal accessory nerve traction injuries. Additionally, the intervention led to improved electrophysiological scores, thereby underscoring the therapeutic value of perioperative ES application.

### 4.2 The mechanism of electrical stimulation therapy for peripheral nerve injury

The mechanism by which ES facilitates recovery in PNI is highly intricate, encompassing a multitude of factors. Principally, to establish a regenerative microenvironment, the majority of nerve stimulators position two electrodes at the termini of the injured nerve, thereby generating an intrinsic electric field at the site of injury. Typically, the anode electrode is situated at the proximal end, while the cathode electrode is placed at the distal end. Regeneration-related factors such as NGF are polarized and electrophoretically migrated toward the cathode under the influence of the built-in electric field. This concentration gradient can guide growth cones of nerves toward the cathode, thereby promoting peripheral nerve regeneration. The intrinsic electric field enhances axoplasmic flow (47), thereby promoting the transport of protein synthesis from neuronal cell bodies to nerve terminals. ES influences the neuron's membrane potential, triggering the opening of Ca^2+^ channels and a subsequent influx of Ca^2+^ ions (48). This influx then activates several proteins, such as adenylyl cyclase (AC), which in turn stimulates the production of cAMP. cAMP enhances the production of growth-associated protein 43 (GAP-43), actin, and Tα-1 tubulin. Concurrently, CREB through the mediation of protein kinase A (PKA), thereby promoting the assembly of cellular scaffolding and facilitating the elongation of growth cones from proximal remnants (49, 50) increased calcium levels can also trigger the upregulation of BDNF and its receptor, TrkB, which collectively influence various downstream effects of ES. Furthermore, an alternative route explains how ES activates CREB via the p38 MAPK signaling pathway (Figure 2B). Research shows that p38 MAPK activation is crucial for BDNF synthesis in the rat hippocampus (51), highlighting the significant function of p38 MAPK in the nervous system, both centrally and PNS.

SCs play a pivotal role in the recovery of PNI. A substantial number of SCs are longitudinally aligned to form Büngner's bands, which facilitate axonal regeneration. Additionally, SCs are capable of forming myelin and secreting neurotrophic factors, thereby establishing an optimal microenvironment for axonal regeneration (52, 53). Nevertheless, the precise function of SCs at these junctions remains to be fully elucidated (54). Earlier studies have shown that the movement of SCs can be influenced by an external electric field (55), with pronounced anodal movement observed at a field strength of 100 mV/mm (56). Investigations employing RNA-seq have examined the differential gene expression between ES and unstimulated rat SCs (55). Research has pinpointed the MAPK pathway as the most notably enhanced pathway during ES, indicating its vital importance in controlling SCs movement. Furthermore, as previously noted, ES activates the CREB via the MAPK pathway, thereby designating SCs and the MAPK pathway as significant research targets for enhancing PNI recovery through ES. The secretion of NGF is a critical function of SCs in facilitating PNI recovery. Researchers (57) have found that ES can enhance NGF release from cultured SCs, with an electric field (1 Hz, 5 V/cm) increasing NGF release by 4.1 times. Furthermore, pharmacological interventions have shown that ES can induce Ca^2+^ influx through T-type voltage-gated calcium channels and mobilize calcium ions from both 1,4,5-trisphosphate-sensitive stores and caffeine/ryanodine-sensitive stores, indicating that ES-induced NGF release by SCs is calcium-dependent. As prototypical glial cells in the PNS, SCs actively remove myelin debris via phagocytosis during the early phase of Wallerian degeneration (58). This process constitutes a critical factor that impedes nerve growth, disrupts the regenerative microenvironment, and obstructs myelin regeneration (59). Therefore, the elimination of myelin debris is essential for nerve regeneration. Li et al. (32, 33) have demonstrated that NGF expedites the disintegration of degenerating nerves and facilitates the removal of myelin debris during Wallerian degeneration. These NGF-mediated effects are modulated via the p75NTR/AMPK/mTOR signaling pathway, which enhances SCs autophagy and promotes autophagic flux. Macrophages also play an important role in regulating their phagocytic activity within damaged lesion regions by secreting inflammatory cytokines and chemokines. McLean et al. (60) found that ES can promote macrophage proliferation and largely polarize them toward an anti-inflammatory M2 phenotype, but the molecular mechanisms involved in this transition are not clear. It has been suggested that (61) increased BDNF levels at lesion sites in spinal cord injury patients could polarize macrophages, and we speculate that a similar mechanism operates in the PNS.

In short, ES aids PNI recovery by enhancing axonal regeneration, influencing neuronal and Schwann cell functions, and boosting nerve growth factor release. While treatments are still being refined, ES presents promising prospects for nerve repair.

## 5 The impact of photobiomodulation on peripheral nerve injury

### 5.1 The application of photobiomodulation in peripheral nerve injury

Photobiomodulation (PBM) is extensively utilized within the field of rehabilitation medicine. Notably, Low-Level Laser Therapy (LLLT) is frequently employed in the management of PNI (8). Lasers can produce powerful, single-color, coherent, and tightly focused light beams with extremely pure frequencies, making them very useful for biomedical applications (62). Sometimes referred to as PBM, constitutes a form of low-intensity light therapy (63). Medical professionals have effectively utilized PBM to treat a variety of challenging health conditions, including non-healing wounds, chronic diabetic ulcers, spinal and nervous system injuries, and chronic pain management (64). In 1987, Rochkind et al. (65) investigated the effects of LLLT on the sciatic nerve in rats. Subsequent research has demonstrated that transcutaneous PBM therapy facilitates the post-traumatic and post-surgical regeneration of injured nerve fibers (66–69). Over the past few years, scientists have more often employed this technique on individuals with nerve damage with LLLT being favored over other physical treatments, especially for carpal and cubital tunnel syndromes (70). In 2014, Fusakul et al. (71) evaluated the efficacy of LLLT in patients with mild to moderate carpal tunnel syndrome and observed significant improvements in the LLLT treatment group compared to the control group, especially in terms of grip strength and electrophysiological assessments. Tascioglu et al. (72) investigated the effectiveness of LLLT for managing carpal tunnel syndrome. Their findings indicated that LLLT did not demonstrate superior effectiveness compared to the control group in terms of symptom improvement, electromyography, and US parameters. This lack of significant difference may be attributed to potential issues related to the selection of laser parameters.

The selection of appropriate parameters is essential for effective treatment, with primary considerations including wavelength, power density, energy density, and the duration of laser application. A review of both animal studies and clinical trials suggests a preference for laser wavelengths in the range of 630–905 nm, predominantly within the infrared spectrum, and energy densities spanning from single to triple digits (70). In 2004, Gigo et al. (73) carried out research to examine the impact of different laser treatments on rats with severed median nerves. The study utilized three types of laser emissions: continuous wave (808 nm), pulsed wave (905 nm), and a combination of both. Through the examination of muscle mass and nerve morphology in muscles innervated by the median nerve, the researchers determined that the combination of pulsed and continuous laser therapy produced the most favorable functional outcomes. Hsieh et al. (74) explored the effects of LLLT on neuropathic pain in rats with sciatic nerve compression injuries, finding that using 660 nm, 9 J/cm^2^ of LLLT significantly reduced the overexpression of HIF-1α, TNF-α, and IL-1β, markedly improved the mechanical withdrawal threshold and the functional indices of the sciatic, tibial, and peroneal nerves, and activated VEGF and NGF. Therefore, they identified LLLT as an innovative clinical approach for ameliorating tissue hypoxia/ischemia and inflammation in compressive neuropathies, as well as for facilitating nerve regeneration. In 2014, Wang et al. (75) conducted a comparative study on the effects of three different energy densities (3, 8, and 15 J/cm^2^) of laser therapy on a rat model of sciatic nerve transection, all with a wavelength of 808 nm, a laser power output of 170 mW, and a power density of 44.7 mW/cm^2^. By examining the functional status, morphological changes, and neuronal growth in rats, researchers discovered that 808 nm LLLT at lower energy densities (3 J/cm^2^ and 8 J/cm^2^) could facilitate post-compression sciatic nerve regeneration.

### 5.2 The mechanism of photobiomodulation in treating peripheral nerve injuries

Laser therapy, a specialized form of PBM, is characterized by its high directivity, brightness, and monochromaticity. Additionally, it exhibits superior coherence due to the uniform frequency and directionality of the light waves (76). Its biological properties encompass photochemical reactions, thermal effects, pressure effects, and electromagnetic effects, each of which are vital in the regulation of biological tissues (77). In the field of medicine, LLLT is frequently employed as an adjunctive treatment during post-operative rehabilitation to facilitate the recovery of muscular, neural, and joint functions, among other physiological processes (78). LLLT is efficiently absorbed by tissues with minimal attenuation, enabling it to modulate DNA activity and ATP production, thus impacting mitochondrial function. The primary mechanism of laser-induced biological modulation involves the activation of COX within the mitochondrial respiratory chain. The energy derived from absorbed photons during laser treatment augments COX activity, thereby enhancing ATP synthesis. The resultant ATP has the potential to reactivate damaged cells and ameliorate metabolic dysfunctions (79). Experiments have shown that LLLT generates mitochondrial Reactive Oxygen Species (ROS), which in turn trigger the redox-sensitive nuclear transcription factor NF-κB (80) (Figure 2C), a protein that regulates immune responses and enhances synaptic plasticity (81). This process facilitates oxygen entry into cells, thereby restoring cellular respiration and generating reactive oxygen species. Furthermore, LLLT has been shown to alleviate pain and inflammation, prevent tissue necrosis, and effectively reduce nervous system degeneration (82).

Analogous to the effects observed with US and ES, PBM has been shown to promote SCs growth. Li et al. (83) investigated the impact of varying output powers of LLLT at constant wavelengths on the regeneration of rat facial nerves. Their findings indicated that the groups subjected to 250 and 500 mW exhibited enhanced proliferation of SCs, whereas the 1,000 mW group demonstrated an increased rate of SCs apoptosis. It was also determined that the 250 mW laser facilitated Akt phosphorylation through the activation of PI3K, thereby inhibiting SC apoptosis mediated by Akt phosphorylation. These findings are consistent with prior research outcomes (84, 85). Similarly, LLLT has been shown to attenuate the levels of inflammatory mediators, a phenomenon that has also been observed in cases of severe injury to the DRG. Chen et al. (86) demonstrated that LLLT markedly alleviated pain and sensitivity to thermal stimuli in rats subjected to DRG compression. Furthermore, LLLT treatment was associated with a significant reduction in the expression levels of the TNF-α and IL-1β. By enhancing the secretion of chemokine ligand 2 (CCL2) in DRG, Oliveira et al. (87) propose that PBM can modulate macrophage polarization and secretion states. This study concludes that 810 nm PBM facilitates the interaction between neurons and macrophages, resulting in a positive feedback network that accelerates nerve repair. Neurogenic inflammation induces the activation of glial cells within the DRG, resulting in the subsequent production of pro-inflammatory cytokines and chemokines. This process contributes to the development of both peripheral and central sensitization (88). In a rodent model of neuropathic pain, Oliveira et al. (87) observed that PBM led to a reduction in the levels of the nociceptive mediator substance P and TRPV1. Additionally, PBM decreased the temperature of the paws and legs. In PNI, PBM may regulate thermoregulation, neurogenic inflammation, and thermal sensitivity. Zhang et al. (30, 31) demonstrated that LLLT attenuated M1 macrophage-specific markers but enhanced M2 macrophage-specific markers in a rat model of spinal cord injury. This finding substantiates that LLLT facilitates the secretion of diverse neurotrophic factors through the activation of the PKA-CREB pathway in macrophages, thereby promoting axonal regeneration. BDNF and NGF play a critical role in neuronal survival and regeneration. In recent studies (89), 632.8 nm irradiation at a power density of 5 mW and an energy density of 10 J/cm^2^ significantly enhanced the expression of BDNF and NGF. This upregulation was observed to commence immediately post-irradiation and persisted, with levels continuing to escalate until day 21.

In summary, LLLT shows promise for treating PNI by boosting mitochondrial function, modulating the immune response, and aiding nerve regeneration. Future improvements in its parameters and protocols could enhance its clinical effectiveness.

## 6 The impact of aerobic exercise on peripheral nerve injury

### 6.1 The application of aerobic exercise in peripheral nerve injury

Axon regeneration in response to aerobic exercise has been reported to be inconclusive. This inconsistency may be attributed to the lack of standardized treatment protocols, particularly concerning variations in the intensity, duration, and timing of exercise initiation. Using rat muscle creatine content and muscle weight, Hines (90) showed that physical activity can accelerate and improve recovery from peripheral nerve paralysis in 1942. Aerobic exercise, a prevalent form of physical activity, has since been extensively investigated for its effects on PNI rehabilitation. In 1974, Herbison et al. (91) conducted a study on neurohistological outcomes in rats, concluding that high-intensity swimming (2 h per day) may not facilitate the repair of reinnervated muscles. They further suggested that engaging in high-load exercise during the early stages of reinnervation could pose significant risks. Clinical applications and translational research pertaining to aerobic exercise have been investigated. Notably, in 2003, Mennen et al. (92) demonstrated that sensory retraining combined with exercise contraction practices following nerve injury surgery in humans led to enhanced functional recovery.

The mode of aerobic exercise, the timing of exercise initiation, and the intensity of exercise constitute significant areas of contemporary research interest. In a 2017 study, Arbat et al. (93) investigated the impact of forced, passive, and voluntary exercise on PNI. Their findings indicated that exercise intensity, rather than the mode of exercise, prevents motor neuron synaptic stripping following axonal rupture. In 2022, Bai et al. (94) conducted a study on the timing of exercise initiation, specifically analyzing the functional recovery of nerve-injured rats subjected to low-intensity, late-initiated treadmill exercise. Their findings indicate that late-initiated treadmill exercise facilitates sensory motor function recovery, prevents muscle atrophy, and promotes nerve regeneration following autologous transplantation repair of long-gap nerve transection. In 2023, Camuzard et al. (95) classified animal models into four distinct groups: continuous exercise, preoperative exercise, postoperative exercise, and no exercise. Their findings indicated that continuous or early exercise markedly enhances functional outcomes by directly influencing muscle innervation, although it exerts minimal effects on nerve regeneration. In 2022, Di Palma et al. (96) conducted a study on nerve-injured rats, categorizing them into groups based on exercise intensity: fast running, slow running, and intermittent running. Moderate-intensity intermittent running significantly enhances functional recovery by promoting axon sprouting and inducing muscle autophagy, thus stabilizing muscle-nerve interactions.

### 6.2 The mechanisms of aerobic exercise on peripheral nerve injury

**Figure 3:**
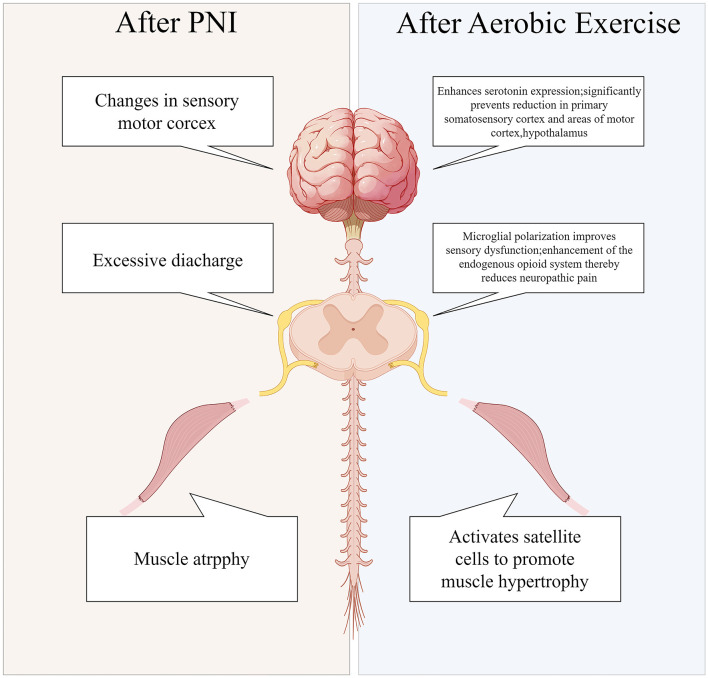
Systemic alterations in peripheral nerve injuries pre-and post-aerobic exercise intervention.

PNI results in a 70% decrease in skeletal muscle cross-sectional area within a 2-month period (97). Furthermore, PNI can result in the atrophy of sensory receptors, leading to subsequent sensory deficits. Additionally, PNI not only leads to localized spinal cord lesions, but it also triggers apoptosis of motor neurons. This neuronal apoptosis is attributed to the impaired transfer of essential nutrient factors to the neuron cell bodies following the injury (98). Lower motor neurons in the spinal cord are less efficient at transmitting information to upper motor neurons in the cerebral cortex as a result of apoptosis of anterior horn motor neurons. Consequently, motor commands originating from the brain fail to reach target organs, such as skeletal muscles, and peripheral sensory information is not conveyed to the brain. This disruption results in impaired cortical sensitivity and motor function (99). EEG findings indicate (100) a reduction in cortical sensitivity among patients with PNI. Aerobic exercise has been demonstrated to enhance blood flow velocity, improve oxygen delivery, stimulate the release of neurotrophic factors, and activate neuronal pathways. Consequently, these physiological changes contribute to systemic improvements in the function of organs, the spinal cord, and the brain (Figure 3), thereby facilitating recovery from injury and enhancing overall functional capacity.

A considerable body of evidence consistently demonstrates that aerobic exercise markedly enhances the neuroimmune response in cases of traumatic PNI (101). Aerobic exercise can (102) significantly shift macrophages from a pro-inflammatory to an anti-inflammatory phenotype, thereby reducing the levels of pro-inflammatory cytokines and alleviating adverse reactions following PNI. Pro-inflammatory cytokines are frequently released at the site of post-traumatic PNI. Findings indicate a significant reduction in the levels of TNF-α following aerobic exercise. These findings indicate that aerobic exercise is pivotal in modulating anti-inflammatory immune responses. Consequently, aerobic exercise exerts substantial anti-inflammatory effects through the regulation of macrophage polarization and TNF-α modulation. As well as providing key insights into the development of targeted rehabilitation programs for PNI, this research provides a theoretical foundation for the application of exercise rehabilitation in the treatment of PNI. Following PNI, BDNF is secreted by activated microglia in the spinal cord. The elevated levels of BDNF subsequently modulate GABA neuronal inhibition, resulting in sensory hypersensitivity (103). Following aerobic exercise, in mice, BDNF expression in the dorsal horn of the spinal cord is reduced (104), which subsequently reverses microglial activation and enhances autophagy flux. Furthermore, as a result of neural injury induced by exercise training, BDNF modulates autophagy via the AKT/mTOR signaling pathway, and polarizes spinal cord microglia (94). In conclusion, exercise training has been demonstrated to effectively mitigate pain-related behaviors in mice. Furthermore, it has been observed that exercise training attenuates the expression of BDNF in the spinal cord dorsal horn following neural injury, thereby reversing sensory hypersensitivity. Additionally, aerobic exercise has been associated with a reduction in levels of GFAP, a marker of astrocytes (105). The promotion of autophagy within glial cells seems to be supported by aerobic exercise. Furthermore, Physical activity has also been shown to enhance serotonin production (106), a neurotransmitter that plays a crucial role in regulating emotions, sleep, and overall brain function, and is also implicated in post-PNI analgesic mechanisms. Despite this, aerobic exercise does not appear to affect BDNF levels consistently across studies. In human research, the majority of experiments indicate that aerobic exercise elevates serum BDNF concentrations (107, 108). Nevertheless, BDNF levels have been reported to be reduced or unchanged following aerobic exercise in a smaller subset of studies (109, 110). The observed trend in BDNF variation appears to exhibit a gender-related pattern. In comparison to control groups, aerobic exercise is associated with an increase in BDNF levels in male patients, while it is associated with a decrease in BDNF levels in female patients (111). Research indicates that testosterone can modulate the expression of BDNF and its receptor, TrkB, in motor neurons. Exercise has been shown to induce a sustained elevation in testosterone levels, which may subsequently result in prolonged upregulation of BDNF and TrkB expression. A similar phenomenon has been observed in ES treatment for PNI (112), where it was found that the related neural regeneration cellular mechanisms require androgen receptor signaling. Empirical evidence from animal studies corroborates these findings, as a result of aerobic exercise, serum BDNF concentrations significantly increased (113–115).

Aerobic exercise significantly impacts PNI rehabilitation by regulating neuroimmune responses, reducing inflammation, enhancing neuroglial autophagy, promoting neuronal activation through increased neurotrophic factor release, and improving blood flow and oxygen supply. Despite some debate over BDNF trends, aerobic exercise is crucial in PNI recovery and supports the creation of personalized rehabilitation programs.

## 7 The application of other physical therapies in peripheral nerve injury

Table 2 summarizes several studies with significant impact on PNI treatment from the four physical methods discussed earlier. When it comes to rehabilitation therapy for PNI, in addition to well-known physical therapies such as US, ES, PBM, and aerobic exercise, new technologies and methods have emerged in recent years that have shown beneficial treatment effects in clinical practice (Table 3).

**Table 2:** The significant studies in each physical therapy modality.

**Physical therapy modality**	**Author and year**	**Experimental content**	**Main conclusion**
Ultrasound	Crisci, 2002	Pulsed US stimulation of proximal nerve stumps for 12 consecutive days.	Identified rapid axonal regeneration with US.
	Tyler, 2008	LILFU effects determined via transcranial US stimulation of hippocampal slice cultures and *ex vivo* mouse brains.	LILFU can non-invasively excite neurons and network activity by activating Na^+^ and Ca^2+^ channels, remotely modulating brain activity.
	Jiang, 2016	Comparison of 250 mW/cm^2^ US intensity treatment vs. 500 mW/cm^2^ or 750 mW/cm^2^ for sciatic nerve injury.	Lower intensity US showed better efficacy for PNI recovery.
Electrical Stimulation	Hoffman, 1952	ES of sciatic nerve at 50–100 Hz after partial L5 nerve root transection in rats.	ES activated neuronal cell bodies in a retrograde manner, enhancing axonal sprouting.
	Brushart, 2002	1 h 20 Hz stimulation in a rat PNI model.	Improved nerve regeneration at junctions; ES initiated motor axon regeneration without accelerating its speed.
	Gordon, 2009	Post-operative ES treatment of median nerve injuries in clinical settings.	Significant improvement in electrophysiological results compared to controls 6–8 months post-treatment.
Photobiomodulation	Rochkind, 1987	Effect of LLLT on sciatic nerve in rats.	Improved electrophysiological outcomes and slower muscle atrophy in the LLLT group.
	Gigo, 2010	Comparison of continuous (808 nm), pulsed (905 nm), and combined laser effects on sciatic nerve in rats.	Pulsed-continuous laser combination promoted better neural morphological and functional recovery.
	Fusakul, 2014	Evaluation of LLLT efficacy in mild to moderate CTS patients with follow-up observations.	The LLLT treatment group showed significant improvements in grip strength and electrophysiological exams.
Aerobic Exercise	Hines, 1942	Division of PNI rats into active and restraint groups.	Activity enhanced recovery speed and extent of peripheral nerve paralysis, and improved muscle atrophy.
	Ariadna, 2017	Exploration of forced, passive, and voluntary exercise effects on PNI.	Exercise intensity was key in preventing synapse detachment in motor neurons post-axonal rupture, rather than exercise type.
	Camuzard, 2023	Division of PNI animal models into continuous exercise, preoperative exercise, postoperative exercise, and sedentary groups.	Continuous or early exercise significantly improved functional outcomes, with minor effects on nerve regeneration.

**Table 3:** An overview of the mechanisms through which various physical therapy modalities facilitate PNI recovery.

**Physical therapy**	**Mechanisms promoting PNI recovery**
Ultrasound	US vibrations activate sensitive ion channels on cell membranes; sound energy converts to heat, generating thermal effects; induces deformation of gas vesicles within cell membranes to affect cellular interactions.
Electrical stimulation	Alters neuron cell membrane potentials to activate Ca^2+^ channels, leading to Ca^2+^ influx, activation of cAMP, further activating CREB, and through the p38 MAPK pathway to activate CREB.
Photobiomodulation	Activates COX in the mitochondrial respiratory chain, thereby increasing ATP synthesis; activates NF-κB.
Aerobic exercise	Enhances blood flow velocity, improves oxygen supply to the body, and can lead to systemic changes including in the brain, spinal cord, damaged nerves, and target organs.
Hyperbaric oxygen	Enhances tissue oxygenation and cellular nutrient production (e.g., creatine phosphate, ATP, and hydroxyproline), promotes formation of new blood vessels, providing optimal conditions for wound healing.
Blood flow restriction	Activates mTOR signaling pathways, regulates synthesis and secretion of muscle growth factors; activates muscle satellite cells.
Magnetic	Accelerates Wallerian degeneration; enhances RNA transcription and protein translation functions of neuronal cell bodies.
Biofeedback (mirror)	Under the action of mirror neurons, cortical areas involved in motor execution can be activated by observing executed movements.

Oxygen is essential for the proper functioning of peripheral nerves, as it is required by nerve cells for energy production within mitochondria (116). Traumatic PNI disrupts the blood supply, thereby diminishing oxygen delivery to the nerves and adjacent tissues. The initial manifestations of local acute ischemia initiate a series of cascade reactions that facilitate angiogenesis (116), inflammation, and cell proliferation. Hyperbaric oxygen therapy (HBOT) augments tissue oxygenation and facilitates the synthesis of cellular nutrients, including phosphocreatine, ATP, and hydroxyproline, thereby promoting angiogenesis and creating optimal conditions for wound healing. Experimental studies have demonstrated that this therapeutic approach is advantageous for peripheral nerve regeneration, attributable to its intrinsic physical properties. Bilsev et al. (117) examined the efficacy of HBOT in patients with upper limb nerve injuries following nerve repair. Their findings indicated superior functional recovery and enhanced electrophysiological outcomes in the HBOT group compared to the control group.

Another therapeutic approach, blood flow restriction (BFR), was employed by researchers who applied a pressure of 130–170 mmHg to the upper arm of a pianist suffering from an upper limb nerve injury (118). A significant increase in forearm and upper arm girth, as well as improved grip strength, was observed after this intervention, following the performance. BFR exercise has the potential to significantly enhance the recovery of residual nerve symptoms in pianists. A critical component of this mechanism is the activation of mTOR signaling (119), which contributes to muscle growth factor secretion and synthesis. Additionally, BFR exercise promotes skeletal muscle hypertrophy through the activation of satellite cells (120).

Emerging evidence indicates that magnetic fields exert a substantial influence on neuronal development. Pulsed magnetic fields (PMF) have been shown to promote nerve regeneration more safely and effectively than static magnetic fields (SMF) (121). Pulsed electromagnetic fields (PEMFs) have been shown to accelerate Wallerian degeneration in distal nerve remnants, thereby creating a conducive microenvironment for the rapid growth of proximal axons into the bands of Büngner. PEMF facilitates RNA transcription and protein synthesis within nerve cell bodies, accelerates the cytoplasmic transport of structural proteins, and promotes axonal regeneration by concentrating neurotrophic factors at injury sites and distal nerve segments. Additionally, PEMF inhibits the formation of collagen fibers, thereby reducing mechanical resistance to axonal regeneration (122). Bademoglu et al. (123) investigated the short-term and long-term electrophysiological and functional regeneration effects of PEMF on sciatic nerve injuries. Their findings indicated significantly greater functional improvements in the intervention groups during weeks 1 and 2 compared to the control groups. However, the effects were not statistically significant in weeks 2, 4, and 6, suggesting that PEMF may be particularly beneficial during the initial stages of recovery (124). Establishing parameters for magnetic stimulation presents significant challenges, given that intense magnetic fields have the potential to harm cellular structures, leading to genetic mutations and DNA damage.

Biofeedback therapy (BFT) presents physiological conditions in readily interpretable visual and auditory formats, thereby facilitating conscious mental control and psychological training to modulate abnormal physiological responses for the regulation of bodily functions, as well as for the prevention and treatment of diseases. In recent years, mirror feedback therapy, a more straightforward variant of biofeedback therapy, has been employed in the management of patients with PNI. The principle posits that cortical regions implicated in motor execution can be activated through the observation of action execution, a phenomenon attributed to the functionality of the mirror neuron system (MNS). This activation has been shown to influence sensory and motor control following PNI (125). Hsu and others (125, 126) conducted a 12-week intervention on patients with injuries ranging from the elbow to the mid-palm. Their findings indicate that mirror therapy may result in superior therapeutic outcomes in terms of hand movement and functional performance for patients with nerve injuries, compared to traditional sensory reeducation treatments. However, it is noteworthy that no significant improvement in sensory function was observed.

## 8 Conclusions and future prospects

An overview of the historical development and therapeutic applications of US, ES, PBM, and aerobic exercise in the treatment of PNI. It delves into the mechanisms of action and their effects on neuroglial cells, NGF, and the immune system. These physical therapies collectively demonstrate the capacity to promote neuroglial cell proliferation, modulate inflammatory responses, and enhance the secretion of NGF. Nevertheless, the signaling pathways through which they facilitate nerve regeneration may differ, necessitating further investigation into their specific mechanisms.

However, each modality of physical therapy presents specific limitations. For example, in the case of US therapy, the variability in soft tissue types, injury patterns, and tissue thicknesses complicates the standardization of its parameters for application in human nerve injuries. And there is a paucity of literature demonstrating the efficacy of US in enhancing sensory function. In addition, the inertial cavitation of high-intensity US may cause irreversible damage to peripheral nerve axons (127). ES for PNI encompasses various methodologies, including traditional approaches such as transcutaneous electrical nerve stimulation (TENS) and implanted ES. TENS is constrained by limited penetration and suboptimal targeting (128), whereas implanted stimulation is associated with high costs and complex surgical procedures. The commonly used 20 Hz low-frequency therapy in clinical practice is ineffective at stimulating deep nerves, despite its ability to activate neuronal activity. In contrast, medium-frequency (1,000–100,000 Hz) electrical stimulation can penetrate soft tissue without causing discomfort. Therefore, interferential current is an ideal option for treating peripheral nerve injuries (129), as it passes through the skin and generates a targeted low-frequency envelope that precisely stimulates the injury site. PBM encompasses intricate irradiation parameters, techniques, methods, and treatment durations, yet it is constrained by limited clinical research. Furthermore, PBM frequently encounters challenges in penetrating human skin, thereby complicating the identification of appropriate phototherapy applications for PNI regeneration and functional recovery (70). The majority of clinical treatments focus on conditions such as carpal tunnel syndrome and cubital tunnel syndrome, which affect the skin's superficial layers. Most previous studies investigating aerobic exercise in the context of PNI have predominantly utilized animal models. Consequently, future research needs to evaluate the applicability of these findings to human subjects. Among PNI patients, particularly those suffering from traumatic injuries, adherence to aerobic exercise regimens remains low. This is further complicated by ongoing debates regarding the optimal parameters and timing of such interventions. Furthermore, physical factor therapies facilitate the promotion of various NGF, such as BDNF, which contribute to the establishment of conducive microenvironments for nerve regeneration. Recent studies suggest that cortical remodeling and somatosensory hypersensitivity following PNI may be associated with these neurotrophic factors (130).

Following nerve injury, spinal dorsal horn BDNF levels are upregulated, enhancing pro-inflammatory responses and pain sensitivity. These observations indicate that the restoration of neurotrophic factor levels via physical exercise may elicit anti-inflammatory effects. Austin et al. (131) observed a significant reduction in spinal BDNF levels in animals subjected to exercise compared to control groups, suggesting that aerobic exercise may confer beneficial effects through the observed decrease in BDNF levels. Consequently, the integration of aerobic exercise with physical factor therapies is recommended. Extensive research has demonstrated that aerobic exercise exerts positive effects on neuroimmunity and analgesia, consistently resulting in reduced inflammation and alleviated symptoms of pain hypersensitivity. Thus, aerobic exercise functions as an intervention to promote early immune responses and mitigate late-stage sensory abnormalities in the treatment of PNI. Integrating various physical therapy modalities can significantly improve treatment outcomes. For example, in cases of long-segment nerve injuries, postoperative swimming training has been demonstrated to enhance upper limb muscle function and volume in rats subjected to 20 mm brachial plexus nerve transplantation (95), although it exerts minimal effects on nerve tissue morphology. Combining aerobic exercise with physical factor therapy has the potential to synergistically enhance functional recovery and nerve regeneration. In a similar vein, addressing the limitations associated with the low penetration of TENS and PBM, we speculate that combining coherent ES with high penetration and US therapy could yield superior results.

Future research can enhance physical therapy for peripheral nerve injury by: (1) optimizing experimental design and standardization to improve research comparability and effectiveness; (2) exploring multimodal therapies, such as combining ultrasound, electrical stimulation, phototherapy, and exercise to boost nerve repair; and (3) conducting in-depth studies on the molecular and cellular mechanisms of nerve repair, focusing on nerve growth factors and inflammation. These efforts will refine therapy strategies and support clinical application.
